# Prognostic significance of circumferential resection margin involvement following oesophagectomy for cancer

**DOI:** 10.1038/sj.bjc.6600931

**Published:** 2003-05-13

**Authors:** O A Khan, J J Fitzgerald, I Soomro, F D Beggs, W E Morgan, J P Duffy

**Affiliations:** 1 Thoracic Unit, Nottingham City Hospital, Hucknall Road, Nottingham, UK; 2 Department of Histopathology, Nottingham City Hospital, Nottingham, UK

**Keywords:** oesophageal cancer, surgery, prognosis, resection margin

## Abstract

The factors affecting long-term survival following oesophagectomy for oesophageal cancer are poorly understood. We examined the significance of microscopic tumour involvement at the circumferential resection margin (CRM) on postoperative survival following oesophagectomy. The case notes of 329 patients who underwent a potentially curative oesophagectomy for squamous or adenocarcinoma were reviewed retrospectively. As part of the procedure, all patients underwent an en-bloc resection of their perioesophageal tissue. The presence of tumour either at, or within, 1 mm of the CRM was recorded and correlated with their TNM and survival data. A total of 67 patients (20%) were noted to have a positive CRM, of which 40 cases (12%) had tumour at the resection margin and the remainder had tumour within 1 mm of the margin. Univariate analysis showed no statistically significant association between survival and either category of CRM involvement. Multivariate analysis showed that only T-stage, nodal status and tumour grade were prognostic markers. In conclusion, the presence of microscopic tumour at the CRM following an en-bloc oesophagectomy is not a significant prognostic marker.

Despite advances in the management of oesophageal cancer, survival following oesophagectomy remains poor with a 5-year survival of approximately 25% (Alexiou *et al*, 1998; Ellis, 1999). A number of studies (Edwards *et al*, 1989; Robey-Cafferty *et al*, 1991; Patti and Owen, 1997) have attempted to identify histological characteristics that correlate with long-term postoperative survival. The presence of microscopic tumour at the circumferential margin of excision is one such histological factor that has recently been investigated as a possible prognostic marker. A retrospective study carried out in 1991 showed that tumour involvement at the circumferential resection margin (CRM) was associated with a higher incidence of local recurrence (Sagar *et al*, 1993). Further work by the same group (Dexter *et al*, 2001) showed that the presence of microscopic tumour at the CRM reduces median postoperative survival; and that the prognostic effect of this factor was most pronounced in those patients with a few metastatic lymph nodes. However, neither of these series examined the long-term prognostic effect of CRM involvement. In addition, both studies were limited by their relatively small sizes. The purpose of our study was to analyse our experience of the significance of microscopic tumour involvement at the CRM on long-term postoperative survival following oesophagectomy in a large cohort of patients.

## METHODS

The case notes of 431 patients who underwent an oesophagectomy for squamous or adenocarcinoma between January 1987 and July 1996 at Nottingham City Hospital were reviewed retrospectively. We then excluded all cases of surgical mortality (defined as death occurring within 30 days of operation), incomplete excision (defined as cases with the presence of microscopic tumour within 1 mm of the proximal or distal margins of excision), primary gastric carcinomas, synchronous tumours and tumours with distant metastases. In addition, we excluded all cases of T4 tumours. These were defined as cases where macroscopic invasion of adjacent structures was noted at operation, as well as all cases where there was microscopic invasion of structures that were resected along with the oesophagus (typically the larynx or pericardium). Following these exclusions, the results of the remaining 329 potentially curative oesophagectomies were analysed in detail. The case notes and operation notes of these patients were reviewed and their survival data were recorded. Patients were followed up for life following their operation, with outpatient clinic review every 3 months for the first year, every 6 months for the next 4 years and yearly thereafter. There was a 100% follow-up rate with a minimum follow-up period among the survivors of 5 years.

All operations were performed by the same team of three thoracic surgeons, who used similar surgical techniques and uniform preoperative management. None of these patients underwent any pre- or postoperative chemotherapy or radiotherapy. All patients underwent a two-field en-bloc oesophagectomy. This entailed local mediastinal dissection with mobilisation of the intrathoracic portion of the oesophagus using diathermy, followed by excision of all perioesophageal tissue with the subcarinal and parahiatal lymph nodes; both parietal pleura overlying the oesophagus and the aortic adventitia. Tumours at the level of the diaphragm were excised together with a cuff of diaphragm. In the abdomen, the lymph nodes from the left gastric artery pedicle were routinely excised, and flush ligation of the left gastric pedicle achieved by application of a vascular stapler.

Pathological analysis was performed by the same team of three histopathologists. On receipt in the laboratory, the surgical specimens were opened avoiding, where possible, the tumour bearing portion of the oesophagus. The specimens were then pinned under gentle tension to a cork board with the serosal surface of the specimen facing upwards. This surface was marked with Indian ink and the specimens were fixed in formalin. The tumours were serially sectioned in the transverse plane and the section with maximal lateral spread of tumour was identified. This section was then embedded and multiple blocks were examined to measure the shortest distance from the outermost part of the tumour to the nearest inked margin. Cases where this distance was less than 1 mm were deemed to have an involved CRM (positive). In addition, a further distinction was made between those cases where the tumour was within 1 mm of the CRM and those where the tumour was present at the margin itself. It should be noted that any case where tumour at the circumferential margin was also within 1 mm of the proximal or distal excision margin was deemed to be a noncurative oesophagectomy and excluded as previously stated. Further analysis was then carried out to determine the histological subtype, T-stage and nodal status of the tumours using the UICC TNM classification (Sobin and Wittekind, 1997). The tumours were also categorised into three grades (namely well, moderately or poorly differentiated) according to the WHO criteria (World Health Organisation, 1977).

Statistical analysis was performed using commercially available software packages. Survival curves for patients with and without CRM involvement were analysed by the Kaplan–Meier method, with differences in survival rates assessed using the log-rank test. The patients' sex, histological tumour type, T-stage, N-stage, tumour grade and CRM involvement were then categorised and a univariate analysis was performed using a *χ*-test to establish any relation between these factors and 5-year survival. Factors that achieved statistical significance (*P*<0.05) on univariate analysis were entered into a multivariate analysis using Cox proportional hazards model to identify independent predictors of survival. Having identified these factors, the effect of CRM involvement on 5-year survival within individual prognostic subgroups was reassessed using a *χ*-test.

## RESULTS

### Patient and tumour characteristics

Of the 329 patients, 218 were male and 111 female with a mean age 65 years (range 28–84). The histological characteristics of the specimens are shown in Table 1

**Table 1:** Distribution of tumours according to tumour type, T-stage, nodal status, degree of differentiation and CRM status (*n*=329)

**Category**	**Number**
*Tumour type*	
Adenocarcinoma	201
Squamous cell carcinoma	128
	
*T-stage*	
1/2	62
3	267
	
*Nodal status*	
N0	133
N1	196
	
*Grade*	
Well/moderately differentiated	144
Poorly differentiated	185
	
*CRM status*	
Positive	67
Negative	262

**Figure 1:**
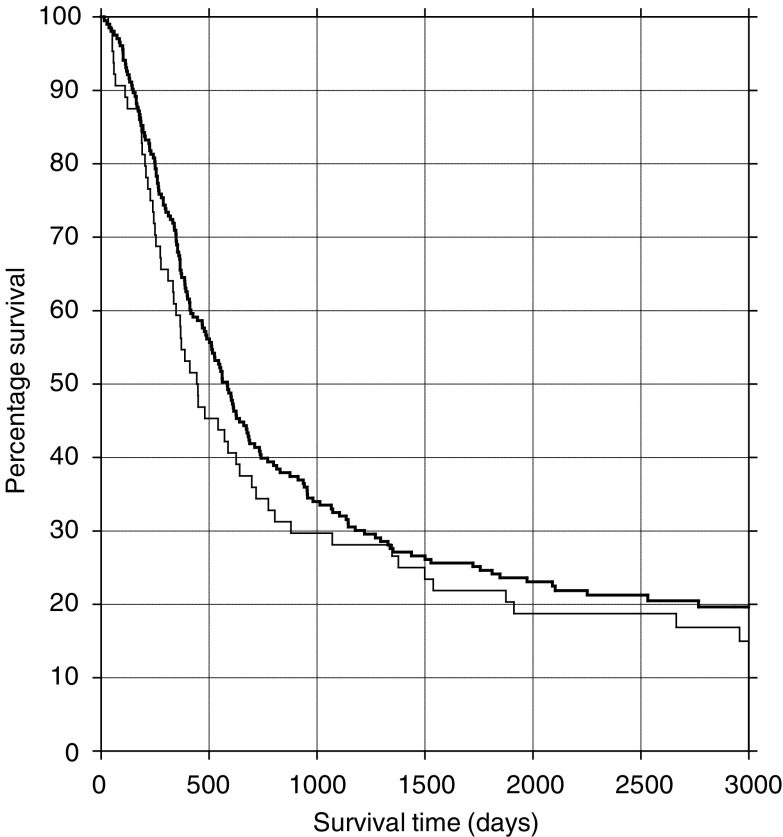
Survival curves for patients with (lower line) and without (upper line) CRM involvement.

. A total of 67 (20%) specimens were found to have microscopic tumour at the CRM. The survival curves of those patients with and without CRM involvement were plotted as shown in Figure 1

. There was no statistically significant difference between the survival pattern of the groups (log-rank test, *P*=0.19). The proportion surviving 5 years in the CRM-positive group was 22% (95% confidence interval 12–32%) and in the CRM-negative group 29% (23–35%). The difference was not significant (*P*=0.25).

### Survival characteristics within the CRM-positive group

Of the 67 CRM-positive cases, 40 were noted to have tumour at the CRM, and 27 had the presence of tumour within 1 mm of the CRM, but not at the CRM. Where the tumour was at the CRM, the 5-year survival was 20% (8–32%) and within 1 mm it was 26% (9–43%). The difference was not significant (*P*=0.57).

### Prognostic factors within the whole population

Univariate analysis was performed to examine the relation between the factors described in the Methods section of this paper and the probability of 5-year survival following oesophagectomy. It should be noted that given the relatively small numbers of T1 and T2 tumours in our population, these were classified as a single subgroup. Similarly, the small number of well-differentiated tumours seen in our population meant that well and moderately differentiated tumours were classified together. The results from the univariate analysis are listed in Table 2

**Table 2:** Univariate analysis of histological characteristics and 5-year survival

**Factor**	**Categorisation**	**Relative risk**	**95% CI**	***P*-value**
T-stage	T1/2 and T3	1.98	1.41–2.79	<0.0001
Nodal status	N0 and N1	9.87	5.27–18.5	<0.0001
Grade	Well/moderately and poorly differentiated	2.00	1.40–2.86	<0.0001
Sex	Male or female	1.26	0.89–1.79	0.20
Tumour type	Squamous or adenocarcinoma	1.26	0.89–1.78	0.19

.

As shown, statistically significant relation with 5-year mortality were found to exist for only three factors: T-stage, grade and number of involved lymph nodes.

Multivariate analysis revealed that T-stage (beta 0.840, hazard ratio 2.32, *P*=0.03), nodal involvement (beta 0.878, hazard ratio 2.41, *P*<0.001) and tumour grade (beta 0.756, hazard ratio 2.13, *P*<0.01) were all independent predictors of survival.

Having established the independent prognostic factors in our cohort of patients, we analysed whether CRM involvement had any effect on survival within particular prognostic subgroups.

### T-stage

No cases of CRM involvement were seen in any patients with T1 or T2 tumours. In view of this, a comparison was made between the 5-year survival of T3 cases with and without CRM involvement. The survival rate for CRM-positive cases was 22% (12–32%) and without CRM involvement it was 24% (18–30%). There was no significant difference between the two groups (*P*=0.69).

### N-stage

Having established that CRM-positive cases occur only in T3 tumours, an analysis of the survival of T3N0 and T3N1 tumours with and without CRM involvement was performed. The survival data are shown in Table 3

**Table 3:** The 5-year survival data for T3 tumours with and without CRM involvement

**Tumour type**	**Survival (**%**)**	**95**% **CI**
CRM positive N0	42	23–61
CRM negative N0	45	33–57
		
CRM positive N1	10	1–19
CRM negative N1	14	8–20
		
CRM positive (W/M)	33	14–52
CRM negative (W/M)	29	19–39
		
CRM positive (P)	16	5–27
CRM negative (P)	20	13–27

N0=nodes clear, N1=nodes involved, W/M=well or moderately differentiated, P=poorly differentiated.

. Univariate analysis revealed that CRM involvement had no prognostic effect in either N0 (*P*=0.84) or N1 (*P*=0.52) disease.

### Grade

The potential prognostic effect of CRM involvement was then reassessed by comparing the survival of T3 well/moderately and T3 poorly differentiated tumours with and without CRM involvement. The survival data of these four groups are shown in Table 3. Univariate analysis revealed that CRM involvement had no prognostic significance in either the well/moderate group (*P*=0.72) or the poorly differentiated group (*P*=0.58).

## DISCUSSION

Although the presence of tumour at the CRM has long been suggested as a potential predictor of survival following oesophagectomy (Skinner *et al*, 1982), it has only been recently that the prognostic significance of this factor has been investigated. Part of the impetus for this research has been the finding that CRM involvement has an important prognostic role in rectal cancer (Quirke *et al*, 1986). Recent studies have shown that CRM involvement in rectal cancer is both a predictor of local recurrence following resection (Adam *et al*, 1994) and a marker of long-term survival (Wibe *et al*, 2002). Given these facts, it is somewhat surprising that only two studies have been conducted in oesophageal cancer to analyse whether an analogous situation exists. Sagar *et al* showed that CRM involvement was associated with decreased median survival, while Dexter *et al* showed that the presence of tumour in the circumferential margin was not only an adverse prognostic factor, but also an independent predictor of survival. The results of our study, however, appear to contradict these findings. Analysis of the survival curves of our CRM-positive and CRM-negative patients showed no statistically significant difference in the short- or long-term outcome. Moreover, this comparison does not even take into account the fact that all our cases of CRM involvement occurred in patients with T3 tumours, a factor which our results show to be an independent predictor of poor prognosis. When this bias is eliminated by analysing the effect of CRM involvement in T3 tumours alone, the situation is even more clear-cut. Within the T3 subgroup, the 5-year survival rates of patients with and without CRM involvement are very similar (22% in the CRM-positive group; 24% in the CRM-negative group). Moreover, we failed to find any statistically significant difference in survival between those patients with tumour at the CRM and those with tumour within 1 mm of the CRM. In addition, we found that CRM involvement did not confer a survival detriment in any prognostic subgroup, unlike Dexter *et al* who found CRM involvement was of particular prognostic significance in patients with N0 disease.

The reason for our differing results is not clear. Although our study differed from that of Dexter *et al*'s in terms of design (ours being a retrospective as opposed to a prospective study), this difference would not, in itself, account for our contrasting results. A partial explanation may lie in our surgical technique of en-bloc oesophagectomy. Although Dexter *et al* do not explicitly state what surgical technique they utilised when performing oesophagectomies, the fact that they reported cases of tumour at the CRM in T2 tumours (where tumour is confined to the muscularis layer) does suggest that they did not perform extensive mediastinal dissection. In addition, their comparatively high rate of CRM-positive cases (64% of their T3 tumours had a positive CRM in comparison to our rate of 25%) further suggests that our patients underwent a more complete resection of their perioesophageal tissue. If this is indeed true, then this raises some interesting questions about the whole question of en-bloc resections. Previous studies (Hagen *et al*, 1993; Altorki *et al*, 1997) have suggested that those patients undergoing an en-bloc oesophagectomy for cancer have an improved survival as compared with those undergoing a more limited resection. In their paper, Dexter *et al* hypothesised that this survival benefit may be a result of improved tumour clearance at the CRM. Although our data would support the notion that en-bloc oesophagectomy reduces the incidence of CRM involvement, we have not shown this reduction to be of any prognostic significance. Interestingly, in the case of rectal tumours, there is some evidence that the prognostic effect of CRM involvement is lessened following radical resections (Merchant *et al*, 1999), and it is possible that a similar situation exists in oesophageal cancer.

It should be noted that the whole concept of the superiority of en-bloc oesophagectomy over more limited resections is by no means certain, and has been an issue of some controversy (Hulscher *et al*, 2001). This question has recently been addressed by a randomised controlled trial (Hulscher *et al*, 2002), which suggested that en-bloc resections confer a better long-term prognosis as compared with transhiatal resection, despite both operations producing similar rates of CRM involvement. Although our study makes no direct contribution to this debate, it does support the notion that any long-term survival benefit seen following an en-bloc resection is not a result of a reduced rate of CRM involvement.

Within our cohort of patients, T-stage, nodal involvement and tumour grade were all independent prognostic factors. This once again contrasts with the results of Dexter *et al* who found that CRM and nodal status were the only significant prognostic determinants. Indeed, the dichotomy between our results and those of Dexter *et al* reflects the somewhat confused nature of the literature on the topic of prognostic markers in oesophageal cancer. Although a large number of studies have been conducted on this subject, there is conflicting evidence as to the prognostic importance of many factors such as T-stage (Patti and Owen, 1997; Dexter *et al*, 2001), tumour type (Dexter *et al*, 2001; Siewert *et al*, 2001) and tumour grade (Edwards *et al*, 1989; Robey-Cafferty *et al*, 1991).

These disparities highlight the importance of utilising standardised preoperative, operative and postoperative techniques on a large population in order to assess prognostic factors – criteria that our study, unlike most previous studies, does satisfy.

In summary, we have shown that following a potentially curative oesophagectomy, the presence of microscopic tumour either at or within 1 mm of the CRM is not a significant prognostic variable. Given the prognostic irrelevance of CRM status, we would question whether this factor should be considered as an essential part of pathology reporting for oesophageal cancer.
